# A pathogenic titin missense mutation in hiPSC-derived cardiomyocytes predisposes to ventricular fibrillation in acute ST-segment elevation myocardial infarction

**DOI:** 10.3389/fcvm.2025.1691585

**Published:** 2026-01-02

**Authors:** Ying-Ying Ji, Yang Wu, Ji Sun, Xiao-Xiong Lin, Xu-Miao Chen, Cheng-Cheng Ji, Li-Juan Liu, Yun-Jiu Cheng, Su-Hua Wu

**Affiliations:** 1 Department of Cardiology, First Affiliated Hospital, Sun Yat-Sen University, Guangzhou, China; 2 NHC Key Laboratory of Assisted Circulation (Sun Yat-Sen University), Guangzhou, China; 3 Department of Cardiology, Guangdong Cardiovascular Institute, Guangdong Provincial People’s Hospital, Guangdong Academy of Medical Sciences, Guangzhou, China

**Keywords:** ventricular fibrillation, ST-segment elevation myocardial infarction, TTN mutation, iPSCs, patch clamp

## Abstract

**Background:**

There is growing evidence that genetic factors may play a crucial role in the development of ventricular fibrillation (VF) following acute ST-segment elevation myocardial infarction (STEMI). Previous studies have demonstrated that both truncating and missense mutations in the TTN gene, which encodes a large cellular structural protein, are linked to arrhythmogenesis. However, the precise role of TTN mutations in the onset of primary VF after acute STEMI remains poorly understood.

**Objectives:**

The primary objective of this study was to comprehensively determine the role and elucidate the electrophysiological mechanism of TTN missense mutations in VF development after acute STEMI. This was achieved by leveraging the unique capabilities of human-induced pluripotent stem cell-derived cardiomyocytes (iPSC-CMs).

**Methods:**

Whole-genome sequencing identified TTN mutations in four out of six unrelated patients who experienced VF after STEMI (STEMI/VF). Induced pluripotent stem cells were successfully generated from the peripheral blood mononuclear cells obtained from a 47-year-old male carrying a novel TTN variant (c.43803T>A/p.D14601E) and from an unrelated healthy control. Subsequently, a directed differentiation protocol was employed to create iPSC-CMs, followed by rigorous electrophysiological and functional analyses to elucidate the mechanisms underlying the observed phenotypes.

**Results:**

Compared with the control iPSC-CMs, the patient-derived iPSC-CMs with STEMI/VF exhibited a significant shortening of the action potential duration (APD). Specifically, peak sodium channel current (I_Na_) and L-type calcium channel current (I_Ca–L_) of the STEMI/VF iPSC-CMs were significantly reduced by 51.64% and 71.56%, respectively. However, the transient outward potassium current remained comparable between the two groups. Moreover, the expression of total TTN in STEMI/VF iPSC-CMs was downregulated. Confocal microscopy analysis revealed that although STEMI/VF iPSC-CMs retained the ability to assemble well-aligned titin striations similar to control cells, there were localized disruptions in the sarcomeric patterning.

**Conclusions:**

The iPSC-CMs obtained from the patient carrying a TTN missense mutation and who suffered from VF after STEMI exhibited a decrease in I_Na_, I_Ca–L_, and shortened APD, providing novel insights into the potential electrophysiological mechanisms underlying the development of VF in the context of acute STEMI and TTN mutations.

## Introduction

Coronary artery disease is estimated to account for approximately 75%–80% of sudden cardiac death (SCD) cases (1). Notably, in 50% of individuals with underlying heart disease, SCD represents their first clinical manifestation (2). Patients with acute myocardial infarction (AMI) are at high risk of SCD, often triggered by ventricular fibrillation (VF). Recent advances in early revascularization therapy for AMI have significantly decreased the incidence of VF and improved survival (3, 4). Nevertheless, approximately 10.0%–11.6% of patients with acute ST-segment elevation myocardial infarction (STEMI) develop VF before acute revascularization (5, 6), with the majority of episodes occurring before hospital arrival. Indeed, the incidence of VF or SCD during this phase is believed to be underestimated, since the exact number of victims of AMI-related SCD before first medical contact remains unclear. Hence, there is a pressing need to identify which individuals with STEMI are at risk for primary VF (PVF). Unfortunately, identifying patients with STEMI at high risk of VF or SCD remains a challenge for clinicians.

Observational studies have shown that individuals with a positive family history of sudden death are more susceptible to the occurrence of SCD or PVF during AMI (6, 7), strongly suggesting the presence of genetic susceptibility. Recent studies have underscored the crucial role of both common genetic mutations and rare variants in genes such as encoding a sodium channel (SCN5A) in the occurrence of VF during AMI (8, 9).

Titin, a giant sarcomeric protein primarily expressed in human striated muscles, is encoded by the TTN gene located on chromosome 2. Structurally, it is comprised of the N-terminal Z-disk, A-band, C-terminal M-line regions, and the elastic I-band domain and is known to play a crucial role in maintaining the structural architecture of the sarcomere, sensing biomechanical stress, and mediating signaling responses. Patients with dilated cardiomyopathy (DCM) who are carrying titin-truncating variants (TTNtvs), the leading genetic cause of DCM, have been shown to exhibit a significantly higher risk of ventricular arrhythmias than non-carriers (10). The pathogenetic role of TTN missense variants (TTNmvs) remains unclear because of the relatively high prevalence of benign TTNmvs in the general population (11). A recent study identified two distinct missense mutations at residue 3892 of TTN, which were respectively associated with familial DCM in two unrelated pedigrees (12). Additionally, several studies have linked TTNmvs with several electrical phenotypes such as atrial fibrillation (13), sick sinus syndrome (14), and electrocardiographic QT interval (15). Hence, TTNmvs have the potential to cause diseases. Titin may affect the action potentials of cardiomyocytes through TTN-binding proteins regulating ion channel function, such as sodium and potassium channels (16–19). Based on previous research, we hypothesized that TTNmvs may increase the vulnerability to VF during acute STEMI through modification of cardiac ion channel function.

Apart from recapitulating the function of human cardiac ion channels of interest, such as heterologous expression systems, human-induced pluripotent stem cell-derived cardiomyocytes (iPSC-CMs) carry the important constituents of human cardiomyocyte ion channel macromolecular complexes essential for normal electrophysiological features. iPSC-CMs have been utilized to mimic the phenotypes associated with a spectrum of cardiac disorders, including both hereditary conditions such as Brugada syndrome (BrS) (20) and acquired cardiac disorders such as diabetic cardiomyopathy (21). Here, we examined the effects of TTN missense mutation using human iPSC-CMs to determine whether its mutation was associated with VF during acute STEMI. Electrophysiological modification of the TTN missense mutation was evaluated by patch-clamp technology.

## Methods

### Clinical features and ethics statement

We collected six unrelated patients with STEMI who experienced VF within the first 6 h after the onset of complaints before primary percutaneous coronary intervention (PCI). Acute STEMI was diagnosed based on the criteria proposed by the third universal definition of myocardial infarction (22). We performed a comprehensive evaluation, including coronary angiography (CAG), echocardiography, electrocardiogram (ECG), Holter monitoring, cardiovascular magnetic resonance (CMR), and other clinical profiles, to exclude structural heart diseases, prior myocardial infarction, and other types of arrhythmias. The investigation was conducted in accordance with the principles formulated in the Declaration of Helsinki and approved by the Institutional Review Board of the First Affiliated Hospital, Sun Yat-sen University. Written informed consent for the study was obtained from all participants.

### DNA sequence analysis

Genomic DNA isolated from peripheral blood samples was used for whole-genome sequencing (WGS). Fragmented DNA samples were processed for the construction of DNA libraries and then amplified by polymerase chain reaction (PCR). The resulting sequencing data were compared against human reference sequences to identify genetic variants. The population frequency of a variant was determined using established genomic databases (e.g., the Genome Aggregation Database and the 1000 Genomes Project). The location and percentage spliced-in score of a mutant locus in TTN were obtained from the CardioDB (https://cardiodb.org/titin).

### Generation of iPSC lines

The iPSC lines were generated from peripheral blood mononuclear cells of a 47-year-old male carrying a TTN missense variant and one ethnically matched healthy subject. As described previously (23), we employed the Sendai virus vector to deliver the transcription factors (e.g., OCT4, SOX2, KLF4, and c-MYC) to reprogram peripheral blood mononuclear cells to pluripotency. At least three independent iPSC lines were generated from each of the two subjects.

### Culture and maintenance of iPSC lines

The iPSC lines were cultured until the logarithmic growth phase was reached. Dissociated with Versene solution (Gibco), the cells were maintained in the mTeSR1 medium (STEMCELL Technologies) at 37°C and 5% CO_2_.

### Differentiation of iPSC-CMs

The iPSC lines were separated and resuspended in the mTeSR1 medium for cardiac differentiation. After cell counting, single-cell pellets were seeded in a 12-well plate at a density of 1.5 × 10^5^ cells per well and cultured for 4 days. After an 85% confluence was reached, the cells were induced for differentiation by treating them with 6 µM CHIR99021 in RPMI + B27 without insulin for 48 h. On day 2, the medium was replaced with RPMI + B27 without insulin and CHIR99021. On days 3–4, 5 μM IWR (Sigma) was added to inhibit Wnt signaling and induce cardiac differentiation. On day 5, the medium was replaced with RPMI medium containing B27 supplement without insulin, and the cells were continually maintained for 2 days. From day 7 onwards, the RPMI medium containing B27 supplement with insulin was used for differentiation until beating cells emerged. Cardiomyocytes were purified using the lactate-based metabolic selection method. The RPMI medium without glucose, containing B27supplement and insulin, was used for culture for 3 days. The methods used for examining the characteristics of iPSC lines and iPSC-CMs are detailed in the Supplementary Material.

### Quantitative polymerase chain reaction (qPCR)

Total RNA was prepared using TRIzol reagent (Invitrogen, USA). RNA concentration and purity were calculated with the NanoDrop 2000 spectrophotometer (Thermo Fisher Scientific, USA) following the manufacturer’s standard instructions. The extracted RNA was separated on an agarose gel to determine structural integrity. Subsequently, 2 µg of total RNA was used for cDNA synthesis using the SuperScript III reverse transcriptase (Invitrogen, USA) with a total reaction volume of 13 µL. Primer sequences of the TTN gene and qPCR amplification conditions are detailed in the Supplementary Material. Gene expression was normalized to GAPDH.

### Immunofluorescence staining and confocal microscopy

iPSC-CMs were fixed with 4% paraformaldehyde at 37℃ for 15 min and permeabilized with 0.5% Triton X-100 for 5 min. After blocking with 2% bovine serum albumin for 1 h, the cells were incubated with an anti-titin antibody (Abcam) (1:200 dilution) at 4°C overnight. The next day, the cells were incubated with the secondary antibody CF 488A goat anti-rabbit IgG (H + L) (Biotium, 1:1,000 dilution) for 1 h at room temperature. DAPI (Thermo Fisher Scientific) was used to stain cell nuclei, and the stained cells were imaged at room temperature using an Olympus SpinSR 10 confocal microscope (Tokyo, Japan).

### Patch-clamp recordings

Spontaneously beating iPSC-CMs were prepared for electrophysiological studies. The electrophysiological recordings of I_Na_, I_Ca–L_, I_to_, and AP parameters were obtained using an EPC-10 amplifier (HEKA Instruments, Lambrecht, Germany) through whole-cell patch-clamp technologies. All electrophysiological measurements were finished at room temperature. AP parameters, including action potential amplitude (APA), resting membrane potential (RMP), maximal upstroke velocity (*V*_max_), and action potential duration (APD) recorded from beat start to 30%, 50%, and 90% repolarization (APD30, APD50, and APD90, respectively) were measured.

Current densities were shown as pA/pF and defined as the current amplitude divided by the membrane capacitance. The (in)activation values of all ion channels were generated by plotting the membrane conductance or relative current against voltage. The activation curves were fitted with the Boltzmann function: *G*/*G*_max_ = [1 + exp(*V*_1/2_-*V*)/*k*]^−1^. The inactivation curves were fitted with the function: *I*/*I*_max_ = [1 + exp(*V*-*V*_1/2_)/*k*]^−1^.*V*_1/2_ indicates the voltage of half-maximal ion channel (in)activation, and *k* is the slope factor. The recovery curves were fitted with the one-phase potential association function: *I*/*I*_max_ = 1 − exp(−*t*/*τ*), where *τ* is the recovery time constant and *t* indicates the time interval between the paired pulses.

### Statistics analysis

All data analysis was performed using GraphPad Prism 9.0 (GraphPad Software). If not stated otherwise, data were presented as mean ± standard error of the mean (SEM). For data comparison between the control and STEMI/VF groups, Student's and Welch's *t*-tests were applied to assess statistical significance. *P* < 0.05 with a two-sided test was considered statistically significant.

## Results

### Clinical data

**Figure 1:**
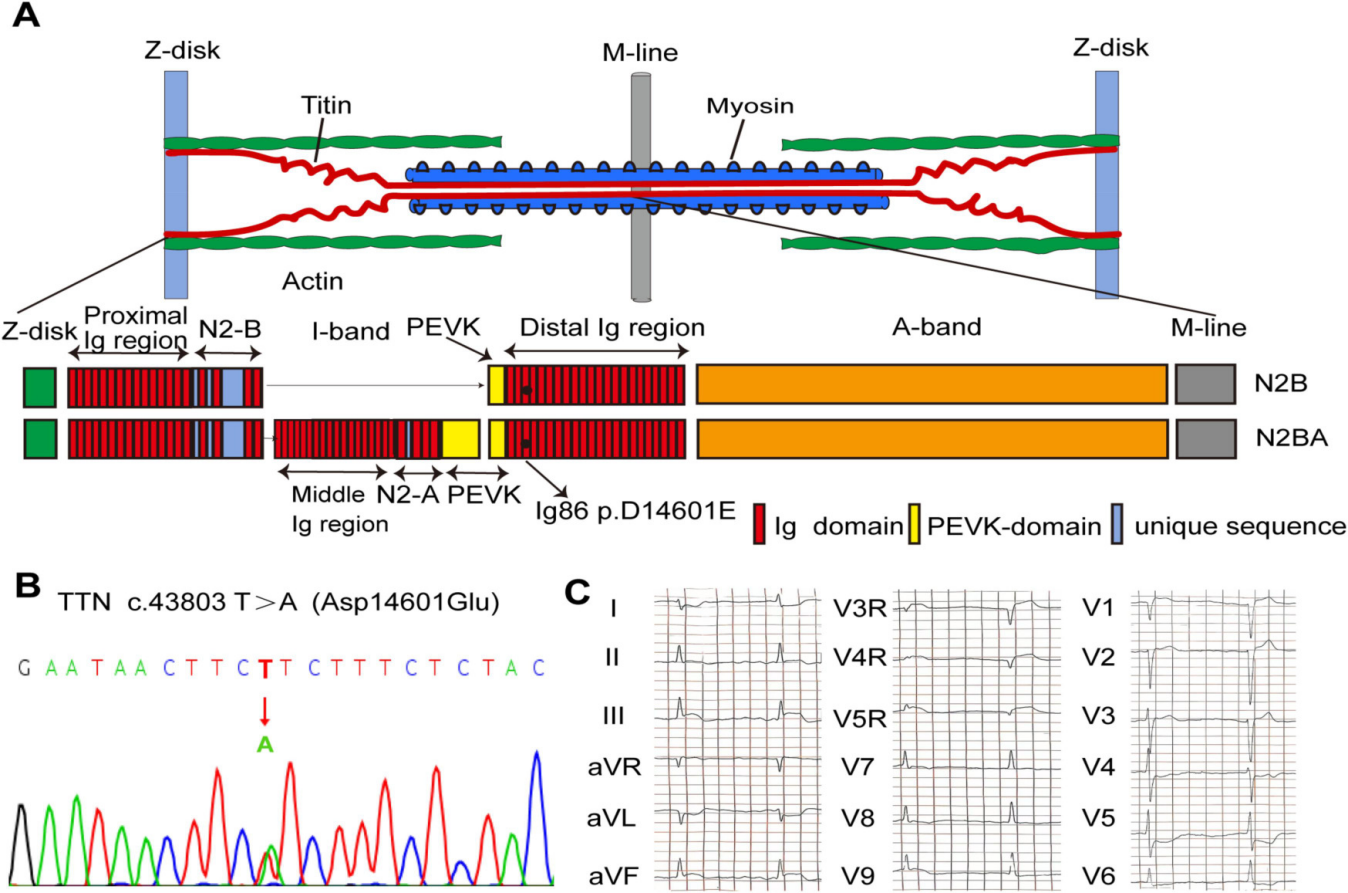
Genetic analysis of the D14601E-titin-positive patient with VF after STEMI. **(A)** Schematic indicating the main components of the human cardiac sarcomere and the location of titin in the sarcomere. Schematic of two main isoforms of titin (TTN) in the human heart, N2B and N2BA, indicating the location of D14601E variant within the Ig86 region of the I-band. **(B)** DNA sequencing trace for the D14601E variant identified in TTN. **(C)** Eighteen-lead ECG before PPCI showing evolving characteristics of inferior and right ventricular STEMI. STEMI, ST-segment elevation myocardial infarction; VF, ventricular fibrillation; Ig, immunoglobulin; ECG, electrocardiogram; PPCI, primary percutaneous coronary intervention.

TTN missense mutations were identified in four of six unrelated patients with sudden cardiac arrest caused by PVF during STEMI. After screening for multiple TTN missense mutations for allele frequencies, protein function prediction, and location, the TTN c.43803T>A/p.Asp14601Glu variant was identified as the suspected pathogenic mutation. The patient enrolled in our study carried a heterozygous missense mutation (c.43803T>A/p.Asp14601Glu) located in the 86th immunoglobulin (Ig)-like domain of I-band in TTN (NM_001267550.1, Figures 1A,B). He experienced recurrent episodes of mild chest discomfort for 2 days and was subsequently transported to the hospital due to sudden cardiac arrest while working one morning, at the age of 47. VF tracings were recorded on the ECG by emergency medical technicians. Immediate cardiopulmonary resuscitation and defibrillation were started. His ECG recordings revealed dynamic changes indicative of inferior and right ventricular STEMI (Figure 1C). In line with these findings, emergency CAG revealed total occlusion of the proximal right coronary artery (RCA), sub-occlusion of the proximal left circumflex branches, and moderate to severe stenosis in the left anterior descending artery. Urgent PCI was performed on the RCA to revascularize the culprit vessel. No episodes of VF occurred during his hospitalization. The results of CMR, echocardiography, and Holter monitoring indicated no signs of structural heart diseases, prior myocardial infarction, or other types of arrhythmias. His family history of SCD and syncope was negative. No episodes of SCD or VF have occurred during a follow-up of 4 years.

### Generation and characterization of iPSC-CM lines

**Figure 2:**
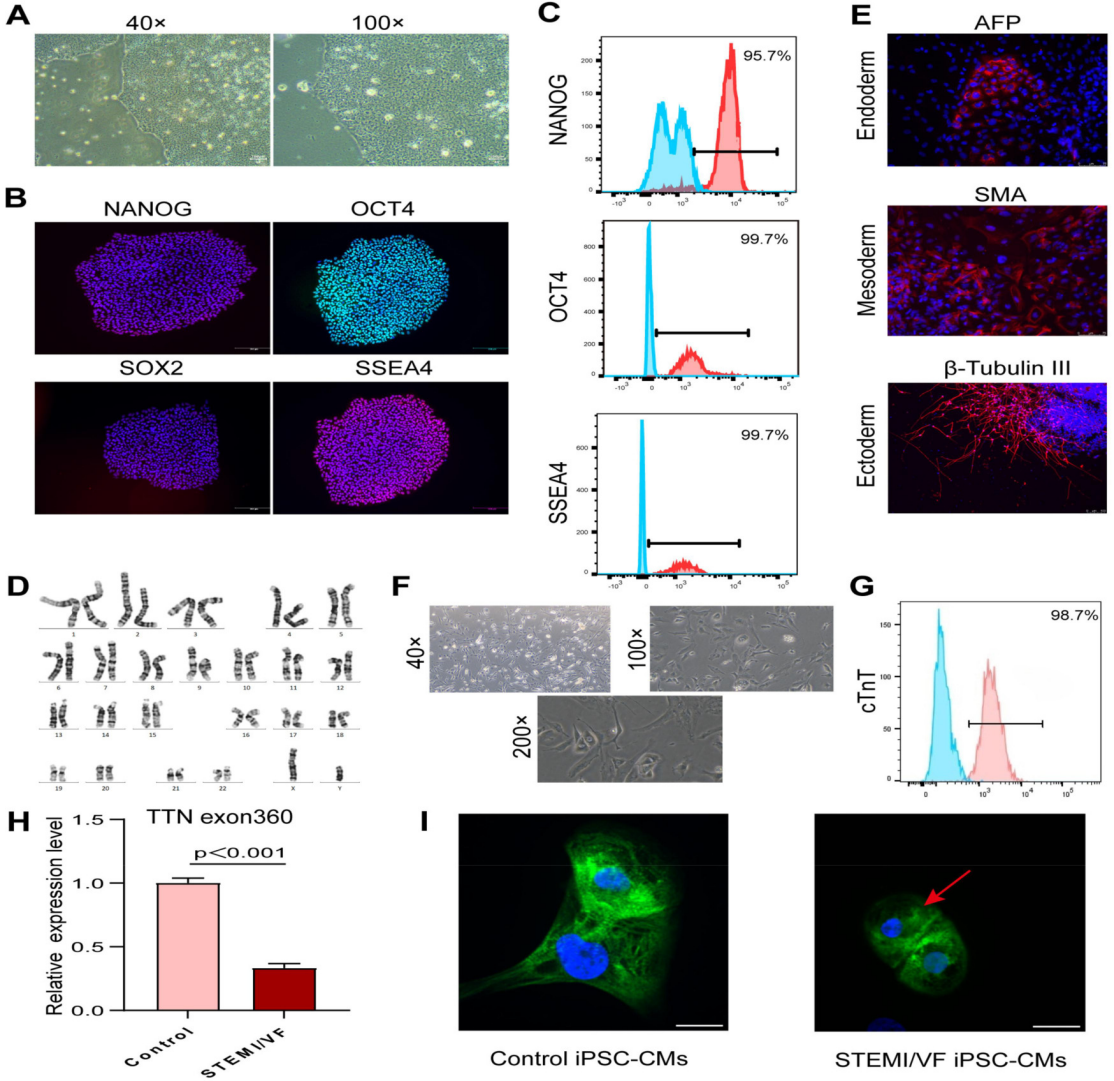
Generation of iPSC-CM lines and analysis of titin expression and organization in iPSC-CMs. **(A)** Brightfield images of the characteristic morphology of iPSCs from the STEMI/VF patient. **(B)** Confirmation of pluripotency of the STEMI/VF iPSC lines by positive staining of pluripotency markers NANOG, OCT4, SOX2, and SSEA4. The nuclei were stained blue using DAPI. Scale bar, 200 μm. **(C)** Flow cytometry analysis of the expression of iPSC markers NANOG, OCT4, and SSEA4. The blue histogram represents the isotype control, while the red histogram represents the test sample stained with the specific antibody. **(D)** G-banding analysis of the karyotype of chromosomes at metaphase in the STEMI/VF iPSCs. **(E)** Confirmation of spontaneous differentiation potential of the STEMI/VF iPSCs by immunostaining of all three developmental germ layers markers AFP, SMA, and β-tubulin III. The nuclei were stained blue by staining with DAPI. Scale bar, 75 μm. **(F)** Typical morphology of derived cardiomyocytes from the STEMI/VF iPSCs using light microscopy. **(G)** Flow cytometry analysis of the percentage of cells staining positive for the cardiac-specific marker cTnT in the derived cardiomyocytes (over 98%). The blue histogram represents the isotype control, while the red histogram represents the test sample stained with the specific antibody. **(H)** The mRNA expression level of TTN was downregulated in the STEMI/VF group compared with the control group (*P* < 0.01, *n* = 3 per group). Student's *t*-test was applied. **(I)** Immunostaining analysis showed that partial disorganized arrays of titin (green) in the STEMI/VF group (red arrow) compared with the control group. The nuclei were stained with DAPI (blue). *n* = 4 per group. iPSC-CM, induced pluripotent stem cell-derived cardiomyocyte; iPSC(s), induced pluripotent stem cell(s); STEMI/VF, ventricular fibrillation after ST-segment elevation myocardial infarction; cTnT, cardiac troponin T. Scale bar, 20 μm. Error bars indicate the standard error of the mean.

The iPSC lines derived from the STEMI/VF patient demonstrated typical human embryonic stem cell morphology (Figure 2A), expressed positive pluripotency markers (NANOG, OCT4, SOX2, and SSEA4) (Figure 2B) by staining, and revealed the expression levels of positive pluripotency markers were over 95% (Figure 2C). The iPSC colonies exhibited normal karyotypes (Figure 2D). Immunofluorescence staining analysis of cell types from endoderm, mesoderm, and ectoderm spontaneously differentiated from iPSCs displayed positive staining for germ-layer markers (AFP, SMA, and β-tubulin III), respectively (Figure 2E). The iPSC lines were hereafter differentiated into cardiomyocytes (Figure 2F). Derived cardiomyocytes were confirmed by staining for the characteristic cardiac markers such as cardiac troponin T with a purity of over 98% (Figure 2G).

### Changes in the expression and organization of titin in STEMI/VF iPSC-CMs

The mRNA expression of total titin was analyzed by qPCR. A significant decrease in the gene expression of total titin was observed in STEMI/VF iPSC-CMs compared with control iPSC-CMs (Figure 2H, *P* < 0.001). Confocal microscopy analysis showed that STEMI/VF iPSC-CMs displayed distinct striated patterns of titin organization, while exhibiting partial disorganization at the cellular level (Figure 2I). Titin within iPSC-CMs from both groups exhibited predominant cytoplasmic localization, with minor nuclear presence observed (Figure 2I).

### AP parameters in STEMI/VF iPSC-CMs

**Figure 3:**
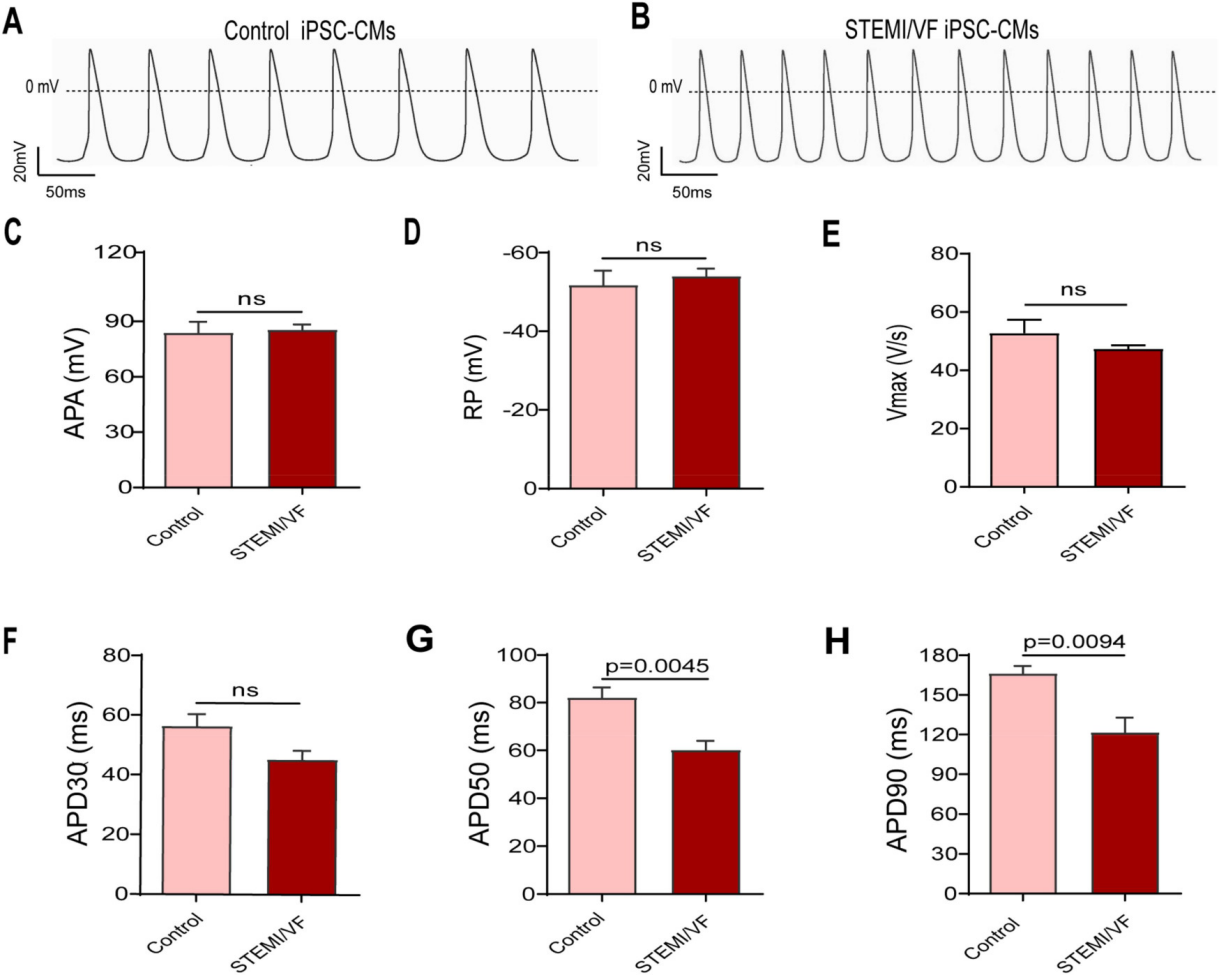
AP traits observed in ventricular-like iPSC-CMs from the STEMI/VF and control groups by single-cell patch clamp. Typical AP traces from the control iPSC-CMs **(A)** and the STEMI/VF iPSC-CMs **(B)**. The differences between the STEMI/VF and control groups were statistically non-significant for APA **(C)**, RP **(D)**, *V*_max_ **(E)**, and APD30 **(F)** (*P* > 0.05). Compared with the control group, APD50 **(G)** and APD90 **(H)** were significantly shorter (*P* < 0.01), *n* = 5 cells for the control group and *n* = 6 for the STEMI/VF group. iPSC-CMs, induced pluripotent stem cell-derived cardiomyocytes; STEMI/VF, ventricular fibrillation after ST-segment elevation myocardial infarction; AP, action potential; APA, action potential amplitude; *V*_max_, maximal upstroke of action potential duration; APD30, APD50, and APD90, action potential duration recorded from beating start to 30%, 50% and 90% repolarization, respectively. Error bars indicate the standard error of the mean. ns, *P* > 0.05. Student's *t*-test was used in all figures except for Figure 3E, where Welch's *t*-test was applied.

AP parameters from the STMEI/VF and control iPSC-CMs were recorded (Supplementary Table S2). The AP pattern was abnormal, and the frequency was faster in STEMI/VF iPSC-CMs compared with control iPSC-CMs (Figures 3A,B). APA, RP, *V*_max_, and APD30 were similar in both cell lines (Figures 3C–F). Compared with control iPSC-CMs, STEMI/VF iPSC-CMs demonstrated significantly shortened APD50 and APD90 (APD50: 60.11 ± 3.92 vs. 82.06 ± 4.33 ms, *P* < 0.01; APD90:121.40 ± 11.33 vs. 166.10 ± 5.82 ms, *P* < 0.01; *n* = 6 for the STEMI/VF iPSC-CMs and *n* = 5 for the control iPSC-CMs) (Figures 3G,H).

### Electrophysiological characterization of I_Na_ in STEMI/VF iPSC-CMs

**Figure 4:**
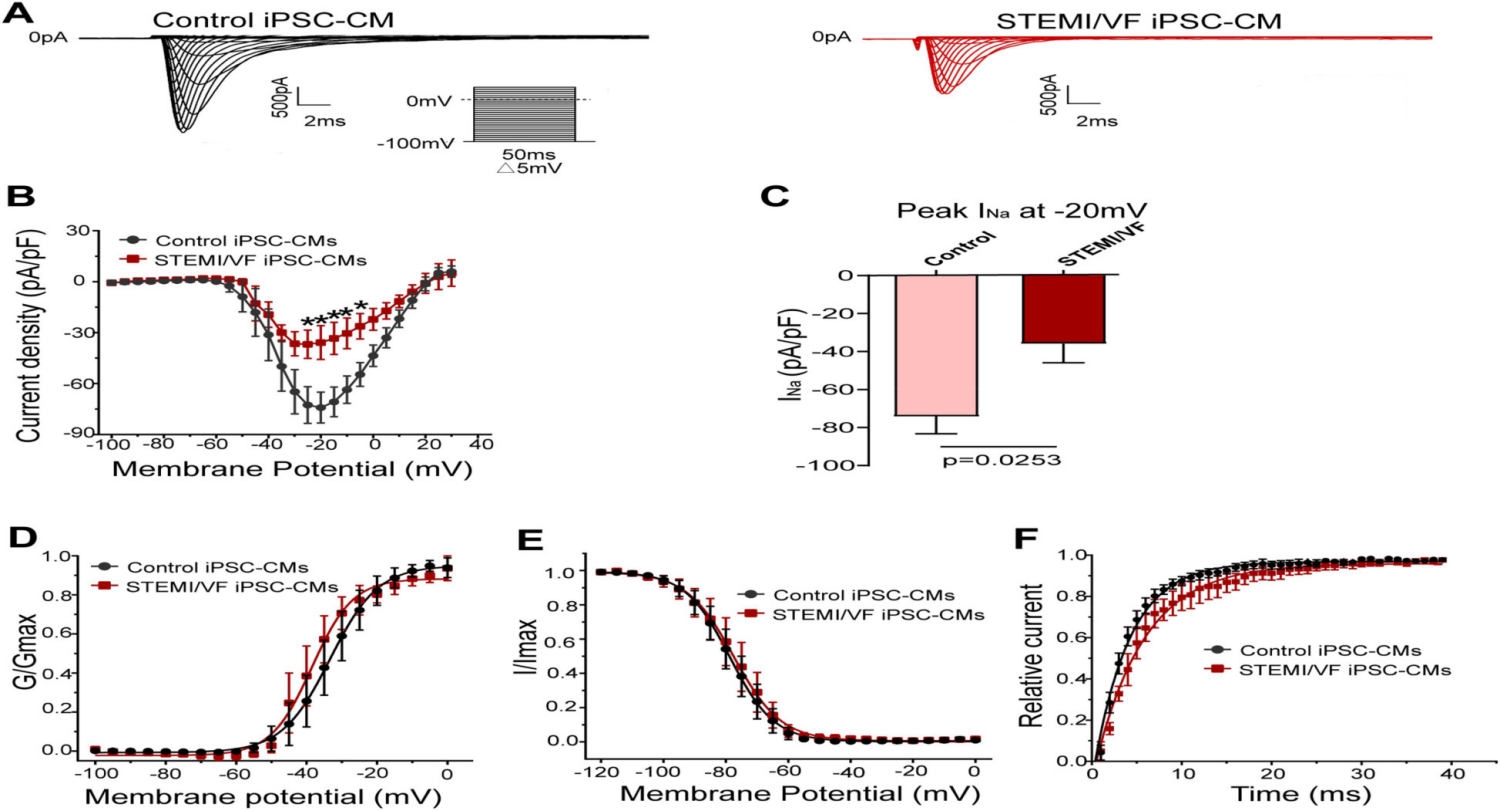
Electrophysiological characteristics of I_Na_ current in iPSC-CMs from the STEMI/VF and control groups. **(A)** Representative I_Na_ traces in the control (left) and the STEMI/VF iPSC-CMs (right). Voltage protocol for activation is shown inset. **(B)** Comparison of the voltage-dependent current density characteristics of I_Na_ in the STEMI/VF (*n* = 4) and control groups (*n* = 5). **(C)** Bar graph showing a significant difference in peak current density of I_Na_ at −20 mV between the STEMI/VF (*n* = 4) and control groups (*n* = 5). **(D)** Voltage-dependent curves for activation (*V*_1/2_: −33.35 ± 1.27 mV, *n* = 5, for the control group vs. −38.49 ± 1.09 mV, *n* = 4 for the STEMI/VF group, *P* < 0.05). **(E)** Voltage-dependent curves of inactivation (*n* = 4 for the STEMI/VF group and *n* = 5 for the control group). **(F)** Voltage-dependent time course curves of recovery from inactivation (*τ*: 6.23 ± 0.90 ms, *n* = 4 for the STEMI/VF group, 4.03 ± 0.33 ms, *n* = 5 for the control group, *P* < 0.05). *P < 0.05. *V*_1/2_, the voltage at half-maximal activation or inactivation; *τ*, the time constant; iPSC-CMs, induced pluripotent stem cell-derived cardiomyocytes; STEMI/VF, ventricular fibrillation after ST-segment elevation myocardial infarction. Error bars indicate the standard error of the mean; Student's *t*-test was used in all figures except for Figure 4B, where both Student's and Welch's *t*-tests were applied.

Typical I_Na_ traces of both groups were presented in Figure 4A. Compared with control iPSC-CMs, I_Na_ amplitude was significantly smaller in the STEMI/VF group. I_Na_ density of the STEMI/VF group was significantly decreased from −25 to −5 mV compared with the control group (Figure 4B). The peak current density of the STEMI/VF group displayed a marked decrease by 51.64% at −20 mV (−35.87 ± 9.96 vs. −74.18 ± 9.09 pA/pF, *P* < 0.05; *n* = 4 for the STEMI/VF group and *n* = 5 for the control group) (Figure 4C). Figure 4D shows the activation curves of both groups. STEMI/VF iPSC-CMs gave a negative shift in the activation curve (−38.49 ± 1.09, *n* = 4) compared with the control cells (−33.35 ± 1.27, *n* = 5; *P* < 0.05). The *k* of activation between the two groups did not differ significantly. There were no statistical differences between the two groups for the *V*_1/2_ and slope factor k of inactivation (Figure 4E). Recovery of I_Na_ from inactivation was slower in cardiomyocytes from the STEMI/VF group (*τ* = 6.23 ± 0.90 ms, *n* = 4) as compared with that from the control group (*τ* = 4.03 ± 0.33 ms, *n* = 5, *P* < 0.05) (Figure 4F). The gating kinetic parameters were listed in Supplementary Table S3.

### Electrophysiological characterization of I_Ca–L_ in STEMI/VF iPSC-CMs

**Figure 5:**
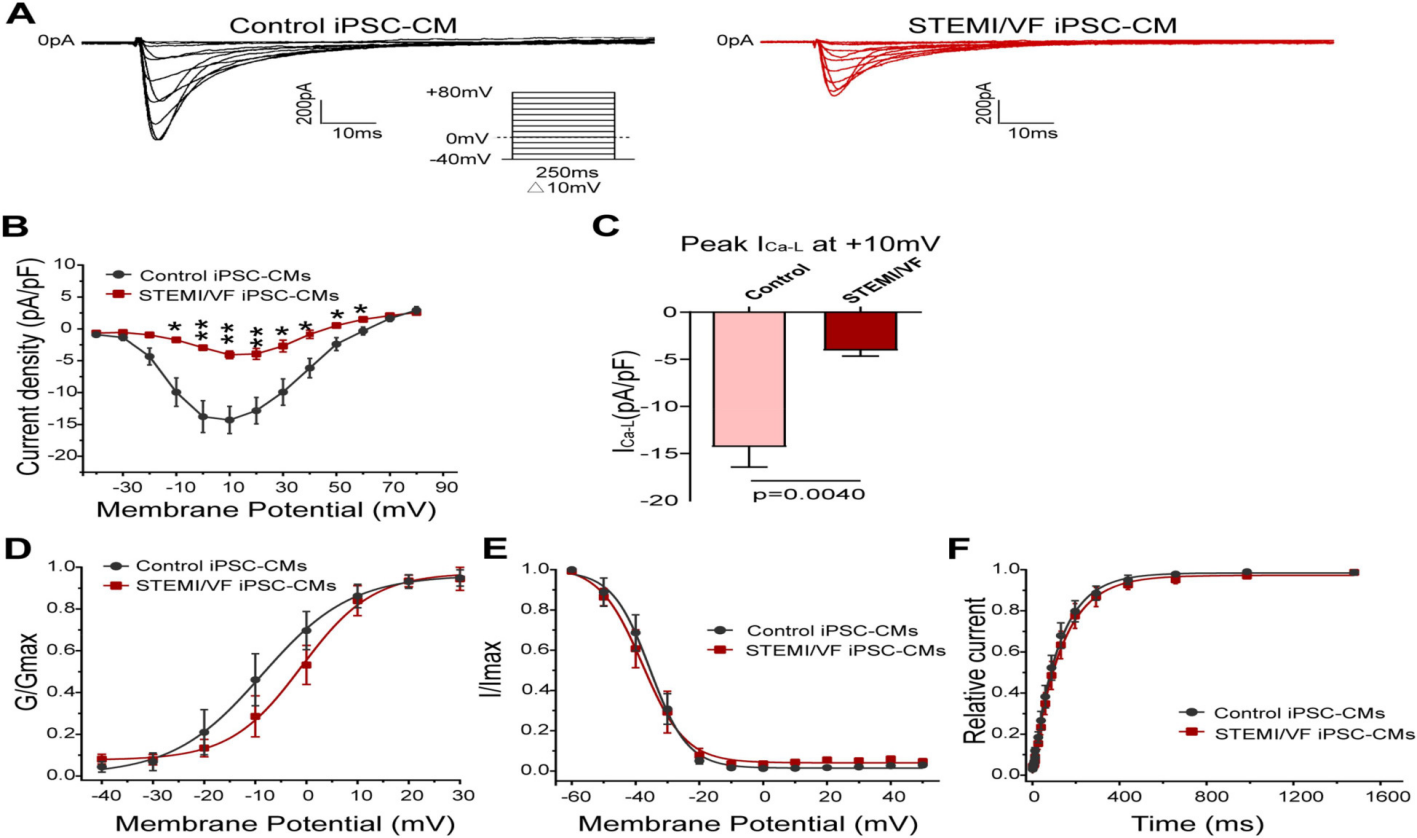
Electrophysiological characteristics of I_Ca–L_ current in iPSC-CMs from the STEMI/VF and control groups. **(A)** Representative I_Ca–L_ traces in the control (left) and the STEMI/VF iPSC-CMs (right). Voltage protocol for activation is shown inset. **(B)** Comparison of the voltage-dependent current density characteristics of I_Ca–L_ in the STEMI/VF group (*n* = 5) and control group (*n* = 6). **(C)** Bar graph showing significant difference in peak current density of I_Ca–L_ at +10 mV between the STEMI/VF and control groups (*n* = 5 per group). **(D)** Voltage-dependent curves for activation (*V*_1/2_: −8.72 ± 2.67 mV, *n* = 5, for the control group vs. −0.95 ± 2.02 mV, *n* = 5 for the STEMI/VF group, *P* < 0.05). **(E)** Voltage-dependent curves for inactivation (*n* = 5 per group). **(F)** Voltage-dependent time course curves of recovery from inactivation (*n* = 5 per group). *P < 0.05, **P < 0.01. *V*_1/2_, the voltage at half-maximal activation or inactivation; iPSC-CMs, induced pluripotent stem cell-derived cardiomyocytes; STEMI/VF, ventricular fibrillation after ST-segment elevation myocardial infarction. Error bars indicate the standard error of the mean. Statistical analyses employed Student's *t*-test for all figures except Figure 5B (both Student's and Welch's *t*-tests) and Figure 5C (Welch's *t*-test).

Figure 5A demonstrates the typical I_Ca–L_ traces of both groups. I_Ca–L_ density of the STEMI/VF group was reduced from −10 to +60 mV compared with the control group (Figure 5B). At 10 mV, the cardiomyocytes of the STEMI/VF group exhibited dramatically reduced peak I_Ca–L_ (−4.07 ± 0.61 pA/pF, *n* = 5) compared with the control group (−14.31 ± 2.13 pA/pF, *n* = 6, *P* < 0.01) (Figure 5C). A positive voltage shift was detected for the activation curve in STEMI/VF iPSC-CMs (*V*_1/2_: −0.95 ± 2.02 vs. −8.72 ± 2.67 mV, *n* = 5 per group, *P* < 0.05) (Figure 5D). Neither the inactivation (Figure 5E) nor the recovery kinetics (Figure 5F) was statistically significant in both cell lines. The characteristics of the gating kinetics of I_Ca–L_ were shown in Supplementary Table S4.

### Electrophysiological characterization of I_to_ in STEMI/VF iPSC-CMs

**Figure 6:**
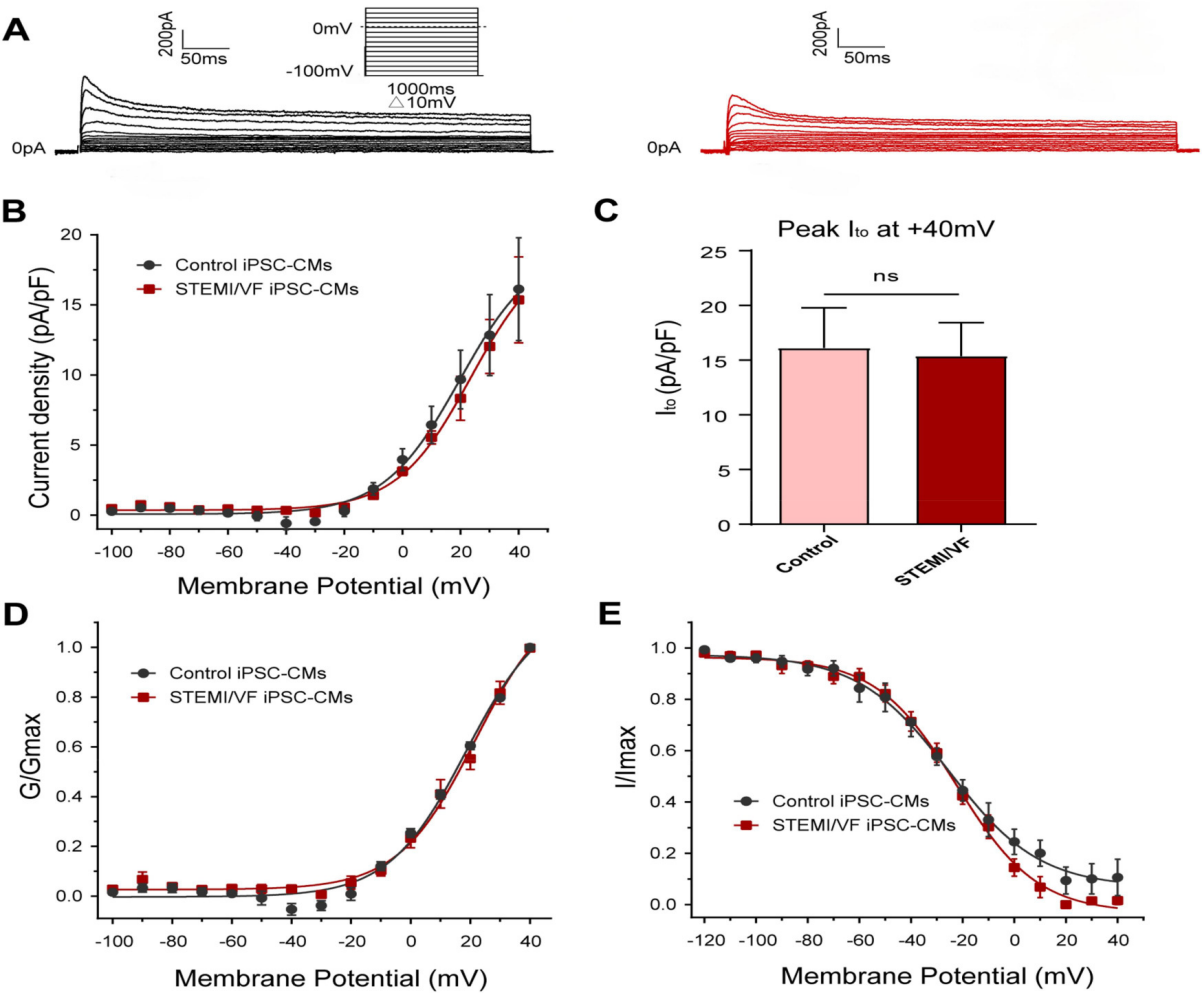
Electrophysiological characteristics of I_to_ current in iPSC-CMs from the STEMI/VF and control groups. **(A)** Representative I_to_ traces in the control (left) and the STEMI/VF iPSC-CMs (right). Voltage protocol for activation was shown above. **(B)** Comparison of the voltage-dependent current density characteristics of I_to_ in the STEMI/VF and control groups (*n* = 6 for the STEMI/VF group and *n* = 5 for the control group). **(C)** Bar graph showing no significant difference in peak current density of I_to_ at +40 mV between the STEMI/VF (*n* = 6) and control groups (*n* = 5) (*P* > 0.05). **(D)** Voltage-dependent curves of activation (*n* = 6 for the STEMI/VF group and *n* = 5 for the control group). **(E)** Voltage-dependent curves of inactivation (*n* = 5 per group). iPSC-CMs, induced pluripotent stem cell-derived cardiomyocytes. STEMI/VF, ventricular fibrillation after ST-segment elevation myocardial infarction. Error bars indicate the standard error of the mean. ns, *P* > 0.05. Student's *t*-test was used in all figures.

Typical I_to_ traces of the two groups were presented in Figure 6A. The I_to_ density was similar in both iPSC-CM lines from the STEMI/VF (*n* = 6) and control groups (*n* = 5, *P* > 0.05) (Figure 6B). There was no significant difference in the peak I_to_ density at +40 mV (15.36 ± 3.07 vs. 16.13 ± 3.67 pA/pF, *P* > 0.05; *n* = 6 for the STEMI group and *n* = 5 for the control group) (Figure 6C). The differences in the gating kinetic parameters of activation and inactivation between the two groups were not statistically significant (Figures 6D,E). Of note, the current was too small to assess the characteristics of recovery from inactivation. The characteristics of the gating kinetics of I_to_ were shown in Supplementary Table S5.

## Discussion

Here, we have, for the first time, established a human iPSC-CM model derived from a STEMI patient with PVF carrying a TTN missense mutation and characterized the electrophysiological properties of iPSC-CMs from the patient. Compared with control cardiomyocytes, electrophysiological experiments revealed that iPSC-CMs from STEMI/VF showed (i) a reduction of I_Na_ and I_Ca–L_ density and (ii) APD50 and APD90. These results demonstrated electrophysiological modification induced by the D14601E variant. Our findings indicated that the structural protein TTN might be implicated in the development of VF in the setting of AMI.

The TTN gene encodes titin with a length varying from 5,604 to 35,991 amino acids. In the human heart, titin serves as a critical regulator of passive myocardial stiffness through its spring-like properties, simultaneously functioning as the principal molecular determinant of diastolic elastic recoil. Loss-of-function variants in TTN are the established genetic cause linked to DCM, and both truncating and missense variants have been linked to arrhythmogenesis in humans (10, 13, 14). Titin interacts with its ligands, including sarcomeric proteins and signal molecules involved in stress-dependent signaling pathways, thereby initiating downstream biochemical events (24). Titin directly or indirectly interacts with a variety of proteins involved in the trafficking and function regulation of cardiac ion channels, such as telethonin (16), ZASP1 (17) and FHL2 (25). The first reported association between TTN and arrhythmias at the ion level was in atrial fibrillation (19), where a nine-amino acid deletion within the A-band Ig-like domain of titin resulted in a gain-of-function of slow delayed rectifier potassium current as well as a shortened APD.

In the present study, we observed a reduction of peak I_Na_ and I_Ca–L_. Loss-of-function of I_Na_ and I_Ca–L_, caused by mutations in SCN5A and calcium channel genes, respectively, has been associated with inherited cardiac arrhythmias, such as BrS (20) and short QT syndrome (26). WGS excluded pathogenic variants in SCN5A or calcium channel subunits as the underlying genetic cause of loss-of-function of I_Na_ and I_Ca–L_. I_Na_ generates the rapid upstroke of the cardiac AP and is responsible for cardiac conduction. In 2007, Hu et al. found that a loss of function in I_Na_ caused by SCN5A mutation was linked to arrhythmic storm during AMI (9). I_Ca–L_ plays a pivotal role in AP generation, propagation, and duration. Inhibition of I_Ca–L_ can accelerate ventricular repolarization by shortening APD, resulting in a predisposition to the development of reentrant arrhythmias (27). During the acute phase of myocardial ischemia, a decreased I_Na_ or I_Ca–L_ or increased outward potassium currents during the early stages of the ventricular epicardial AP can give rise to enhanced spatial repolarization dispersion, resulting in markedly elevated ST segments and increased susceptibility to the development of reentrant arrhythmias. Given the distinct electrophysiological bases of ventricular and atrial tissues, we think that this mutation predisposes individuals predominantly to VF, rather than to atrial fibrillation. Although the I_to_ remained unchanged, we observed significantly decreased inward currents. Thus, the D14601E TTN missense mutation was expected to predispose to ischemia-related VF by affecting the function of I_Na_ and I_Ca–L_. The shortened APD can be explained, at least in part, by the reduced I_Na_ and I_Ca–L_. Previous studies suggested that TTN mutations could cause cytoskeletal disarray, altered Nav1.5 localization at intercalated discs, aberrant sodium channel activities, as well as impaired excitation and conduction (16, 18). In addition, it may cause defective excitation–contraction coupling by impairing the trafficking of calcium channels through disrupted interactions with Z-disc and T-tubule proteins, resulting in reduced I_Ca–L_. Thus, the impact of the D14601E mutation on the reduction of I_Na_ and I_Ca–L_ might be indirect and be assumed mainly by disturbing ion channel trafficking. Further studies should delineate the exact molecular mechanisms of the observed reduction in I_Ca–L_ and I_Na_ currents caused by the TTN variant, such as through western blot and qPCR. Notably, the patient had no family history of SCD or arrhythmia-related cardiac events, experienced VF for the first time only in the setting of STEMI at the age of 47, and had no episodes of acute ischemia events or SCD during follow-up, which supported the TTN missense variant as a subclinical pathogenic substrate for acquired arrhythmic syndrome.

The D14601E mutation is located in the Ig86 module of the I-band. Previous studies have demonstrated that point mutations in Ig modules of human cardiac titin are characterized by reduced protein domain stability, which may impair its function (28). Recently, two studies have identified pathogenic missense variants (C3575S and T2850I) within the I-band region of titin (29, 30). These variants disrupted structural domain stabilization, leading to loss of protein function and subsequent cardiomyopathies. Notably, the precise molecular mechanisms, whereby missense mutation-induced destabilization of titin's Ig domains leads to downstream pathological manifestations, have yet to be fully elucidated. Based on previous research, the effect could be mediated via multiple possible mechanisms, such as enhanced susceptibility to titin haploinsufficiency and degradation (as observed for TTNtvs) (31), disrupted interactions between titin domains and their cognate signaling molecules (32), sarcomeric incorporation (33), aberrant posttranslational modifications (31), or synergistic interactions among these mechanisms. We observed ion channel dysfunction and a decreased expression of total titin mRNA in STEMI/VF iPSC-CMs. Moreover, confocal microscopy demonstrated disrupted parallel alignment of titin arrays in STEMI/VF iPSC-CMs compared with control cells. Therefore, we hypothesize that the difference in expression level and partially disorganized arrays of titin caused by a missense mutation may be implicated in the occurrence of ischemia-related VF. Further studies should delineate the molecular mechanisms underlying TTN missense mutation-induced dysregulation of titin expression and sarcomere integration defects.

It should be noted that human iPSC-CMs exhibit immature electrophysiological characteristics compared with native adult cardiomyocytes, particularly in terms of ion channel expression profiles that are critical for normal AP properties. For instance, the I_to_ densities observed in both experimental groups demonstrated a modest reduction compared with adult cardiomyocytes, making it difficult to measure the channel current. Clustered regularly interspaced short palindromic repeats technology was not employed to correct and repair the mutated gene sites at the iPSC level. Future work must validate these electrophysiological observations with an expanded panel of cell lines to confirm these findings and enhance their generalizability. Further comprehensive experiments will be needed to better examine the role of this TTN missense variant in the pathogenesis of ischemia-associated VF, utilizing a novel human *in vitro* model of acute myocardial ischemia constructed by patient-derived iPSCs.

In conclusion, we identified aberrant phenotypic features of STEMI iPSC-CMs carrying a TTN missense mutation that may explain the higher vulnerability of this patient to VF. This study supports the role of titin as a critical molecular integrator connecting sarcomeric structure with cardiac ion channels. Our findings may not only advance our understanding of structural protein pleiotropy in arrhythmogenesis but also highlight titinopathies as potential therapeutic targets for VF associated with ischemia. Future investigations in arrhythmia susceptibility under stress conditions could further strengthen mechanistic insights of the TTN variant in arrhythmogenesis.

## Data Availability

The original contributions presented in the study are included in the article/Supplementary Material, further inquiries can be directed to the corresponding author/s.
