# Supplementary material for: A pathogenic titin missense mutation in hiPSC-derived cardiomyocytes predisposes to ventricular fibrillation in acute ST-segment elevation myocardial infarction
Source: Front Cardiovasc Med. 2026 Jan 2;12:1691585. doi: 10.3389/fcvm.2025.1691585 (PMC12808434; doi:10.3389/fcvm.2025.1691585)
Supplement: Supplementary file 1 [file Datasheet1.pdf]

Supplemental Materials

Supplemental methods

Identification of the pluripotency and normal karyotype of iPSC-CMs

Karyotyping

iPSC lines were pre-treated with colchicine for 2hours, and then enzymatically dissociated for 3-5 min to obtain single-cell suspensions. After hypotonic treatment and centrifugation, cells were fixed with 1ml methanol-glacial acetic acid (3:1). Chromosome preparation was performed by G-band staining. At least 30 metaphases were examined and karyotype was analyzed for 6 metaphases of each iPSC line.

Flow cytometry

Dissociated with TrypLE Express (Gibco) for 15 min, cells were centrifuged and then resuspended in ice-cold methanol (90%) on ice for 30 min. After washed 3 times with 1X Phosphate Buffered Saline, cells were incubated with appropriate primary antibodies at room temperature for 30min.The primary antibody were OCT4 (1:400, Cell Signaling Technology, #2750), NANOG (1:400, Cell Signaling Technology, #3580), SSEA-4 (1:5000, Abcam, #ab16287) and cardiac Troponin T (cTnT) (1:500, Abcam, # ab209813). Cells were labeled with appropriate secondary antibodies, including Alexa fluor 488 conjugated donkey-anti-rabbit IgG(H+L) secondary antibody (1:100, Thermo Fisher Scientific, #A-21206) and Alexa fluor 488 conjugated Goat-anti-mouse IgG(H+L) secondary antibody (1:200, Thermo Fisher Scientific, #A28175) at room temperature for 30 min. Cell sorting was performed on BD FACS Aria II (BD Biosciences) ,and data was read and analyzed using FlowJo software (FlowJo, LLC).

Immunofluorescence staining

The pluripotency markers of iPSCs were identified using immunofluorescence. The primary antibodies included NANOG (Cell Signaling Technology), OCT4 (Santa Cruz), and SOX2 (Cell Signaling Technology), and SSEA4 (Abcam) antibodies. The second antibodies included Alexa Fluor 488 Donkey anti-Mouse IgG antibody (Thermo Fisher Scientific), Alexa fluor 594 Donkey anti-Rabbit IgG antibody (Thermo Fisher Scientific), and Alexa fluor 594 Donkey anti-Mouse I-gG antibody (Thermo Fisher Scientific). DAPI was used to stain cell nuclei.

Quantitative polymerase chain reaction (qPCR)

Primers for each gene were listed in the Supplemental Table 1. The condition for amplification was listed as follows: 95 °C for 30s (pre-denaturation), 40 cycles of 95 °C for 15s(denaturation) and 60 °C for 30s (annealing/extension).

Patch clamp recordings

The extracellular solution for AP parameters contained (in mmol/L):137 NaCl, 4 KCl, 10 HEPES, 10 D-Glucose, 1 $MgCl_2$•$6H_2O$, 1.8 $CaCl_2$•$2H_2O$ (pH 7.4 with NaOH). The pipette solution contained (in mmol/L):135 KCl, 2 $MgCl_2$, 1EGTA, 10 HEPES,4 Mg-ATP, 0.3 Na_2-GTP (pH 7.4 with KOH).

For I_{Na} recordings, the extracellular solution was the same as that for AP parameters. Pipette solution filling the electrodes contained (in mmol/L): 50 CsCl, 60 CsF, 10 HEPES, 20 EGTA,

10NaCl (pH 7.2 with CsOH). With a holding potential maintained at –100 mV, I_{Na} currents were evoked by using depolarizing pulses stepping from –100 mV to 30 mV for 50ms (5-mV increments). The steady-state inactivation protocol involved 500-ms conditional pulses from –120 mV to 20 mV in 5-mV increments with a holding potential at –100 mV, followed by 20-ms test pulses to –20 mV. To measure the time constants of the recovery, a double-pulse protocol was applied with the potential maintained at –120 mV. A 10-ms pre-pulse to –10mV was followed by a 50-ms test pulse to –10mV. The time interval between each paired pulse ranged from 1 to 40 ms in 1-ms increment.

The bath solution for $I_{Ca\text{-}L}$ recordings contained (in mmol/L): 140 TEA-Cl, 5D-Glucose, 4 KCl, 10

HEPES, 1 $MgCl_2 \cdot 6H_2O$, 10 $CaCl_2 \cdot 2H_2O$ (pH 7.4 with TEA-OH). Pipette solution contained (in mmol/L):110 CsCl, 5 HEPES,10 EGTA, 4 Na_2-ATP, 4.5 Mg-ATP, 1 $CaCl_2 \cdot 2H_2O$ (pH7.2 with Cs-OH). For activation, the holding potential was set at –40 mV. iPSC-CMs were depolarized by 250-ms voltage pulses from –40 mV to 80 mV (10-mV increments). With a holding potential at –40 mV, the inactivation of $I_{Ca\text{-}L}$ was measured using 3000-ms depolarizing voltage pulses from –60 mV to 50 mV (10-mV increments) followed by 30-ms test pulses to 10mV. To assess the recovery from inactivation, a double-pulse protocol was applied with a holding potential at –40 mV. A 250-ms pre-pulse was used to depolarize cells to 10 mV, followed by a recovery pulse of –80mV with variable duration from 1 to 20ms (1-ms increment). A 200-ms test pulse to 10mV was then applied.

The extracellular solution for I_{to} currents was the same as that for AP parameters. The pipette solution contained(in mmol/L): 4 NaCl, 10 HEPES, 1 EGTA, 138 K-gluconate, 2 Mg-ATP, 1 $MgCl_2 \cdot 6H_2O$, 0.1 $CaCl_2 \cdot 2H_2O$ (pH7.4 with NaOH). Depolarizing voltage pulses were used to evoke I_{to} ranging from –100 mV to 40 mV (10 mV increments), with a holding potential at −100 mV. For steady-state inactivation of I_{to}, currents were elicited by 1-s conditional pulses from –120 mV to 60 mV in 10-mV increments followed by 500-ms test pulses to 40mV when the membrane potential was maintained at –100 mV.

Supplemental Tables

Supplemental Table 1. The primer sequences used for qPCR

Primer	Forward (5'-3')	Reverse (5'-3')
TTN (exon360)	GCTGGATCTGTGTCCTCTAGC	TCCTCTTGGACAACAGCTTTCT
GAPDH	GTCTCCTCTGACTTCAACAGCG	ACCACCCTGTTGCTGTAGCCAA

Supplemental Table 2. AP parameters of the Control and STEMI/VF iPSC-CMs

Groups	APA (mV)	RP (mV)	Vmax (V/s)	APD30 (ms)	APD50 (ms)	APD50 (ms)
Control iPSC-CMs	**83.76±6.11**	**-51.71±3.72**	**52.76±4.55**	**56.29±4.08**	**82.06±4.33**	**166.10±5.82**
STEMI/VF iPSC-CMs	**85.41±2.95**	**-53.96±1.99**	**47.35±1.19**	**45.19±3.07**	**60.11±3.92****	**121.40±11.33****

**P < 0.01 vs the Control. Values are presented as mean ± SEM. APA, action potential amplitude; RP, resting membrane potential; Vmax, maximal upstroke velocity; APD30, APD50, and APD 90, APD recorded from beat start to 30%, 50%, and 90% repolarization, respectively.

Supplemental Table 3. Gating kinetic parameters of I_{Na} in Control and STEMI/VF iPSC-CMs

Groups	Activation			Inactivation			Recovery	
	$V_{1/2}$(mV)	k	n	$V_{1/2}$ (mV)	k	n	τ(ms)	n
Control iPSC-CMs	**-33.35 ± 1.27**	**6.83 ± 1.11**	**5**	**-78.79 ± 0.87**	**-7.15 ± 0.76**	**5**	**4.03 ± 0.33**	**5**
STEMI/VF iPSC-CMs	**-38.49 ± 1.09***	**5.82 ± 0.95**	**4**	**-77.50 ± 1.07**	**-7.58 ± 0.94**	**4**	**6.23 ± 0.90***	**4**

*P < 0.05 vs the Control. Values are presented as mean ± SEM.$V_{1/2}$, the voltage at half-maximal activation or inactivation; k, the slope factor. τ, the time constant. iPSC-CMs, induced pluripotent stem cell–derived cardiomyocytes. STEMI/VF, ventricular fibrillation during ST-segment elevation myocardial infarction.

Supplemental Table 4. Gating kinetic parameters of I_{Ca-L} in Control and STEMI/VF iPSC-CMs

Groups	Activation			Inactivation			Recovery	
	$V_{1/2}$(mV)	k	n	$V_{1/2}$ (mV)	k	n	τ(ms)	n
Control iPSC-CMs	**-8.72 ± 2.67**	**8.69 ± 2.54**	**5**	**-35.47 ± 1.03**	**-5.92 ± 0.85**	**5**	**132.20 ± 30.41**	**5**
STEMI/VF iPSC-CMs	**-0.95 ± 2.02***	**6.96 ± 1.88**	**5**	**-37.83 ± 1.47**	**-6.74 ± 1.20**	**5**	**143.90 ± 28.06**	**5**

*P < 0.05 vs the Control. Values are presented as mean ± SEM.$V_{1/2}$, the voltage at half-maximal activation or inactivation; k, the slope factor. τ, the time constant. iPSC-CMs, induced pluripotent stem cell–derived cardiomyocytes. STEMI/VF, ventricular fibrillation during ST-segment elevation myocardial infarction.

Supplemental Table 5. Gating kinetic parameters of I_{to} in Control and STEMI/VF iPSC-CMs

Groups	Activation			Inactivation		
	$V_{1/2}$ (mV)	k	n	$V_{1/2}$ (mV)	k	n
Control iPSC-CMs	**18.63 ± 1.97**	**12.94 ± 1.13**	**5**	**-25.36 ± 2.55**	**-17.58 ± 2.42**	**5**
STEMI/VF iPSC-CMs	**21.53 ± 2.90**	**12.86 ± 1.52**	**6**	**-22.38 ± 1.38**	**-15.64 ± 1.25**	**5**

Values are presented as mean ± SEM.$V_{1/2}$, the voltage at half-maximal activation or inactivation; k, the slope factor. iPSC-CMs, induced pluripotent stem cell–derived cardiomyocytes. STEMI/VF, ventricular fibrillation during ST-segment elevation myocardial infarction.
