# Supplementary material for: A pathogenic titin missense mutation in hiPSC-derived cardiomyocytes predisposes to ventricular fibrillation in acute ST-segment elevation myocardial infarction
Source: Front Cardiovasc Med. 2026 Jan 2;12:1691585. doi: 10.3389/fcvm.2025.1691585 (PMC12808434; doi:10.3389/fcvm.2025.1691585)
Supplement: Supplementary file 2 [file Table1.docx]

**Supplementary Patients Materials**

**Table 1. Clinical details of 6 unrelated patients with VF during STEMI**

| **Patient ID** | **Gender** | **Age** | **Location of VF onset** | **STEMI location** | **Culprit artery** | **Number of diseased**  **coronary arteries** | **Family history of SCD and syncoe** |
| --- | --- | --- | --- | --- | --- | --- | --- |
| Patient 1 | M | 47 | Prior to hospital admission | Inferior and right ventricular | RCA | 3 | Negative |
| Patient 2 | M | 59 | Emergency department | Anterior | LAD | 3 | Negative |
| Patient 3 | M | 60 | Prior to hospital admission | Inferior and right ventricular | RCA | 1 | Negative |
| Patient 4 | M | 50 | Prior to hospital admission | Extensive anterior | LAD | 1 | Negative |
| Patient 5 | M | 71 | Emergency department | Extensive anterior | LM | 2 | Negative |
| Patient 6 | M | 49 | Emergency department | Anterior and high lateral | LAD | 3 | Negative |

Patient 1 was the subject of this research. RCA, right coronary artery; LAD, left anterior descending; LM, left main coronary artery.

**Table 2. Genetic analysis of TTN missense variants in 6 STEMI patients with VF**

| **Patient ID** | **Nucleotide** | **Amino acid change** | **dbSNP** | **gnomAD allele frequency** | **Exon** | **Band** | **Percent spliced in (PSI)** |
| --- | --- | --- | --- | --- | --- | --- | --- |
| Patient 1 | c.43803T＞A | p.Asp14601Glu | - | - | 237 | I-band | 100 |
| Patient 2 | - | - | - | - | - | - | - |
| Patient 3 | c.36830C＞A | p.Ala12277Glu | - | 0.000089 | 175 | - | 1 |
| Patient 4 | c.104989C＞G | p.Leu34997Val | rs771364302 | 0.000073 | 358 | M-band | 100 |
|  | c.99102G＞C | p.Trp33034Cys | rs397517778 | 0.000268 | 354 | A-band | 100 |
|  | c.42947－18A＞C | - | rs766977954 | 0.000024 | IVS232 | - | - |
|  | c.39616C＞T | p.Pro13206Ser | rs186404793 | 0.00066 | 208 | I-band | 10 |
| Patient 5 | - | - | - | - | - | - | - |
| Patient 6 | c.49197C＞A | p.His16399Gln | - | - | 262 | A-band | 100 |

Patient 1 was the subject of this research. IVS, intron variant or sequence.
